# Medical Department of Cincinnati College—Edifice—Trustees

**Published:** 1836

**Authors:** 


### II. Medical Department of the Cincinnati College.—Edifice—Trustees.

We have stated in a former number, that the Cincinnati College was chartered on the 21st of January, 1819. It was founded by citizens of Cincinnati. Several hundred contributed to the erection of its edifice. Before this was finished, from reverses of business, those who had expended liberally upon it, were compelled to withhold further contributions, and in 1825, the exercises of the College were suspended. The building went into rapid deterioration, and 10 years afterwards when the Trustees resolved on reviving education within its walls, it was almost a heap of ruins. Its bones, however, were not affected with *frangilitas* nor *caries*, and it was only necessary to restore the soft parts. A rapid regeneration of these, followed the organization of the Medical and Law Departments, but the winter came on before the work was accomplished. Suspended for several months, it has recently been resumed ; and, the better to fit the edifice for the accommodation of the Medical School, a new wing 30 by 45 feet is now in the progress of erection, and will be finished by the first of June. The Faculty will then be in the occupancy of the following departments all fitted up in the most permanent and commodious manner.—1. A lecture room for the non-demonstrative branches, 30 by 42 feet : 2. An anatomical and surgical theatre of the same size : 3. A chemical amphitheatre, of equal dimensions : 4. A laboratory 24 by 30 feet: 5. An anatomical museum of the same dimensions :

6. A dissecting room 30 by 45 feet, with sky lights, a hydrant, vault and all other conveniences. Thus, in reference to apartments, no medical institution in the Union will afford better accommodations, for any number of pupils not exceeding 300. And as to chemical apparatus, anatomical preparations—both healthy and morbid, and subjects for practical anatomy, the whole will be found ample.

The Trustees have also enclosed the grounds about the edifice, and are engaged in planting them with such species, native and exotic, as will constitute a small Botanic Garden; fitted for the illustration of the elements of the science.

After accommodating, in the amplest manner the Medical and Law Departments, the capacious and repaired edifice, will afford an adequate number of large class and recitation rooms, for another school; and the Trustees are now deliberating on the best mode of establishing a department for the education of professional Teachers, in those branches that will fit them to become instructors in the Academies and Colleges of the West.

The Board of Trustees are elected annually by the contributors to the building. It is well known, that as students of medicine came to the city last fall, they were told, that at the election in the ensuing March, the contributors would displace the Board which had established the Medical and Law Schools, and elect one that would abolish both. This was accordingly attempted. The enterprise was planned with care and caution, and, as the disclosures on the day of election demonstrated, every practical effort had been made to render it effective.

But the DESTRUCTIONISTS were utterly defeated. They numbered but *eight*—the CONSERVATIVES more than *one hundred and twenty!* Thus the contributors have sustained the Board, and sanctioned the establishment of the new departments, by an expression, that has rebuked and silenced forever, the few interested individuals, who sought the arrest and overthrow of an institution, which must rapidly advance into public favor abroad, as it already has done throughout the city.

